# Partial Replacement of Cassava Starch With Peruvian Maca Flour in Mortadella: Innovation in Functional Meat Products With Clean‐Label Potential

**DOI:** 10.1111/1750-3841.70314

**Published:** 2025-06-06

**Authors:** Natália da Silva Leitão Peres, Leticia Cabrera Parra Bortoluzzi, Flávia Aparecida Reitz Cardoso, Renata Hernandez Barros Fuchs, Nathália Letícia Hernandez Brito, Sahra Gadia Trelha, Leila Larisa Medeiros Marques, Anielle de Oliveira, Evandro Bona, Adriana Aparecida Droval

**Affiliations:** ^1^ Department of Food Engineering and Chemical Engineering Federal University of Technology ‐ Paraná (UTFPR) Campo Mourão Brazil; ^2^ Post‐Graduation Program of Food Technology (PPGTA) Federal University of Technology ‐ Paraná (UTFPR) Campo Mourão Brazil; ^3^ Post‐Graduation Program of Technological Innovations (PPGIT) Federal University of Technology ‐ Paraná Campo Mourão Brazil

**Keywords:** cassava starch, mortadella, Peruvian maca flour, physicochemical properties, sensory analysis

## Abstract

This study explored the partial substitution of cassava starch with Peruvian maca flour in mortadella formulations, aiming to develop a healthier, clean‐label meat product by increasing antioxidant activity and reducing synthetic additives. Formulations with varying proportions of cassava starch and maca flour were evaluated for their physicochemical properties. Significant differences were observed in pH, water‐holding capacity (WHC), and color, with 100% cassava starch yielding the highest WHC and 100% maca flour producing a more intense yellow hue (higher b* value). Based on optimization, the 25% cassava starch and 75% maca flour formulation were selected for shelf‐life analysis. Over 90 days, this formulation maintained stable pH (6.43 ± 0.02), WHC (90.7 ± 0.5%), and color parameters (L*, a*, b*), and lipid oxidation remained below the critical TBARS limit of 2.0 mg MDA kg^−1^, demonstrating good oxidative stability (1.38 ± 0.04 mg MDA kg^−1^ at 90 days). Color differences (Δ*E* ≈ 2.5) were perceptible but did not compromise consumer acceptance. Flash profile sensory analysis found no significant differences in sensory attributes between samples with and without synthetic antioxidants. Consumer testing using a 9‐point hedonic scale yielded average scores above 7.0 for appearance, flavor, and overall acceptance, indicating high acceptance. These results support using maca flour as a natural antioxidant and functional starch substitute, offering a viable strategy for cleaner‐label, health‐oriented meat products.

## 1 Introduction

Mortadella is a widely consumed cured meat product with significant economic importance in the meat industry's cold‐cuts sector. Its popularity in Brazil began with the arrival of Italian immigrants around 1900. Initially associated with lower socioeconomic classes, mortadella gradually gained market acceptance, becoming a well‐appreciated and commercially widespread food item across various social classes (Cunha et al. 2022). Its broad appeal is due to its unique flavor, soft texture, and versatility in culinary applications, making it a staple in both traditional and modern diets.

The processing of mortadella involves the creation of a stable emulsion that combines meat, fat, and other ingredients such as starches and seasonings. This mixture is stuffed into natural or artificial casings and subjected to thermal treatment to ensure product safety and stability. Depending on specific identity and quality standards, the product may also undergo smoking, depending on the specific identity and quality standards required for commercialization (Molina et al. 2024). Among the ingredients used, starches play a critical role in water retention, texture, and emulsion stability, directly influencing yield and consumer acceptability (Carvalho et al. 2021). Nevertheless, cassava starch (CS) presents limitations such as limited antioxidant activity and a relatively simple nutritional profile, which may reduce its contribution to shelf life or functional labeling.

Despite technological advances, the shelf life and overall quality of these cured meat products can be compromised by lipid oxidation, one of the primary mechanisms of deterioration (Soro et al. 2021). Lipid oxidation leads to the formation of off‐flavors and rancid odors, adversely affecting sensory quality (Wang et al. 2023). Furthermore, it generates potentially harmful compounds, raising concerns regarding food safety and nutritional value (Dragoev 2024). To mitigate these effects, synthetic or natural antioxidants are often employed to stabilize lipids and prolong product shelf life.

Growing consumer demand for clean‐label and functional foods has led the meat industry to increasingly turn to natural additives that act as preservatives and offer potential health benefits. In this context, clean‐label refers to formulations with minimal artificial additives, aligning with consumer demand for more transparent ingredient lists. Functional foods are those that provide additional health benefits beyond basic nutrition, such as antioxidant or anti‐inflammatory properties. Current research emphasizes the incorporation of plant‐based ingredients and functional compounds, such as polyphenols, flavonoids, and dietary fiber, which have been shown to improve oxidative stability while contributing to the nutritional quality of meat products (Ching et al. 2022; Ismail et al. 2020; Shahidi and Ambigaipalan 2015). Andean roots and tubers like Peruvian maca (*Lepidium meyenii*) have gained attention due to their promising bioactive profiles.

Peruvian maca is a tuberous plant native to the high Andes of Peru and Bolivia. It is traditionally consumed for its nutritional and therapeutic properties and has recently been used in functional food formulations. Chemically, maca is rich in carbohydrates (54.6 to 60.0 g 100 g^−1^), proteins (23.02 to 38.48 g 100 g^−1^), and dietary fiber (8.23 to 9.08 g 100 g^−1^), with low lipid content (1.09 to 2.2 g 100 g^−1^) and relevant levels of minerals (4.9 to 5.0 g 100 g^−1^ ash) (Bower‐Cargill et al. 2022; Li et al. 2017). In addition to its nutritional value, maca contains macamides, glucosinolates, and other phytochemicals with demonstrated antioxidant activity, which may contribute to lipid stabilization in meat matrices (Gonzales 2012; Zhang et al. 2020).

Several studies have also pointed to maca's functional properties, including its potential to improve mood, reduce stress, and support memory function (Da Silva Leitão Peres et al. 2020; Kasprzak et al. 2018; Ulloa del Carpio et al. 2024), making it an attractive ingredient for health‐conscious consumers. While previous studies have incorporated ingredients like flaxseed, chia, or quinoa flour into meat emulsions to enhance functionality, these often require masking of off‐flavors or result in undesirable textures. In contrast, maca flour may offer a balance between sensory acceptability and antioxidant potential, making it a promising clean‐label alternative for starch substitution. Moreover, its natural pigments and fiber content may influence the color and texture of food products, which are critical quality parameters in meat processing.

Considering the growing demand for healthier, functional, and additive‐free meat products, this study aims to develop and optimize a mortadella formulation in which CS is partially replaced by Peruvian maca flour. Given the complex interactions between ingredients and their effects on physicochemical and sensory parameters, a mixture design approach is especially suited for identifying optimal combinations. An experimental mixture design was employed to determine the optimal proportion of both ingredients, balancing physicochemical performance with functional benefits. The selected formulation was evaluated over a 90‐day storage period through physicochemical analyses (pH, water‐holding capacity, color, and lipid oxidation) and sensory evaluation using the Flash Profile method. This integrative approach seeks to assess the technical viability of maca flour as a substitute in emulsified meat products and its impact on consumer perception and sensory acceptability.

## 2 Methodology

This study comprised two stages: (i) formulation optimization of mortadella blends incorporating CS and Peruvian maca flour, and (ii) storage stability and antioxidant comparison of the optimized formulation over 90 days. All ingredients were sourced locally (Campo Mourão, PR, Brazil); seasonings and additives were donated by IBRAC Aditivos e Condimentos (BR), and maca flour by Jasmine Alimentos (BR).

### 2.1 Experimental Design and Formulation Optimization

A two‐component mixture design (Barros Neto et al. 2007) varied CS and maca flour across seven formulations (0%–100 % replacement) (Table 1). Formulations F5‐F7 (50:50) served as triplicate central‐point runs to estimate experimental variance. Levels were chosen based on preliminary trials demonstrating stable emulsions and exploring partial and total starch replacement.

**TABLE 1:** Experimental design: Proportions of cassava starch and maca flour used in each formulation.

Experiments	Cassava starch (%)	maca flour (%)
**F1**	0	100
**F2**	100	0
**F3**	25	75
**F4**	75	25
**F5**	50	50
**F6**	50	50
**F7**	50	50

Each batch consisted of 62.5% pork meat, 15% pork fat, 12% ice, 5% starch/maca blend, 2% textured soy protein, 0.5% curing salt (Cura IBRAC, BR), 0.25% sodium tripolyphosphate (ACORDINI#701 Extra, IBRAC, BR), 0.5% seasoning, 1.8% NaCl, 0.1% garlic powder, and 0.1% monosodium glutamate. For positive‐control variants, 0.25 % sodium erythorbate (IBRACOR 501, IBRAC, BR) was included. Ingredients were weighed on a semi‐analytical balance, emulsified in a bowl cutter (Confrimaq, MADO Garant, BR), stuffed (Confrimaq, AISI 304 L, BR) into artificial casings, cooked in a water bath to 68°C core, and cooled under running water for 15 min.

#### 2.1.1 Microbiological Safety

Microbiological analyses were performed on all formulations to verify hygienic‐sanitary quality by Brazilian Normative Instruction No. 62 (Brasil 2003). The following microorganisms were investigated: coliforms at 45°C (log CFU g^−1^), coagulase‐positive *Staphylococcus* (log CFU g^−1^), sulfite‐reducing *Clostridium* spp. (log CFU g^−1^), and *Salmonella* spp. (presence/absence in 25 g). These tests were conducted using standard protocols for enumeration and detection recommended by Brazilian regulations. Although essential to ensure the safety of samples used in sensory and storage evaluations, microbiological quality was not used as an optimization parameter, as contamination risk is primarily linked to processing hygiene rather than formulation composition.

#### 2.1.2 Sensory Acceptance Test

A consumer acceptance test was conducted to evaluate the sensory preference of the mortadellas produced in the first stage of the study. A total of 85 untrained assessors, aged between 18 and 60 years, participated in two evaluation sessions (morning and afternoon) held on two consecutive days, due to the large number of samples (seven formulations, including three repetitions of the central point from the factorial design). Ethical approval for the study was granted under CAAE 88330918.6.0000.5547, and all participants signed an informed consent form.

The sessions took place from 8:30 am to 12:00 pm and from 2:00 pm to 5:30 pm, with mandatory participation of the same assessors in both sessions to ensure consistency. Samples were cut into cubes of approximately 1.5 cm per side, coded with three random digits, and presented individually in randomized order, along with water for palate cleansing.

Assessors were asked to rate five attributes, aroma, color, flavor, texture, and overall impression, using a nine‐point hedonic scale (1 = disliked extremely; 9 = liked extremely). The collected data aimed to determine the overall consumer preference for each formulation and identify the most accepted combinations of CS and maca peruana flour.

#### 2.1.3 Optimization Criteria

The experimental design considered the proportions of CS and maca flour (M) as independent variables. The selected response variables included pH, water‐holding capacity (WHC), color parameters (L*, a*, b*), and overall sensory impression, the latter used as the primary criterion, as it encompasses global consumer perception of the product.

Second‐order polynomial models were fitted for each response variable, using the following general equation (1).

(1) $$y=\beta_{0}+\beta_{1}x_{1}+\beta_{2}x_{2}+\beta_{12}x_{1}x_{2}+\beta_{11}{x_{1}}^{2}+\beta_{22}{x_{2}}^{2}$$

where *y* is the predicted response, *x*_1_ and *x*_2_​ are the coded levels of CS and M, respectively, and the *β*∖beta*β* coefficients are estimated from experimental data.

The optimization of ingredient proportions was based on a desirability function approach (*D*), which allows multiple response variables to be integrated into a single numerical criterion. This approach includes individual desirability functions (*d_i_*), defined for each response variable depending on the optimization goal (maximize, minimize, or target):

(2) $$d_{i}=\left\{\begin{array}{c} 0\\ {\left(\frac{y_{i}\;-\;L_{i}}{T_{i}\;-\;L_{i}}\right)}^{s}\\ 1 \end{array}\begin{array}{c} \mathrm{se}\;y_{i}\leq \;L_{i}\\ \mathrm{se}\;L_{i}\leq y_{i}\leq T_{i}\;\\ \mathrm{se}\;y_{i}\geq \;T_{i} \end{array}\right.$$

where $L_{i}\;$ is the lower acceptable limit, $T_{i}\;$ is the target value, *s* is a weighting factor (commonly 1), and $y_{i}\;$ is the observed value for response *i*.

The overall desirability (*D*) is calculated as the geometric mean of the individual desirabilities (Equation 3).

(3) $$D={\left(d_{1}\cdot d_{2}\cdot \text{…}\cdot d_{n}\right)}^{1/n}$$

All calculations were performed using Statistica 13.0 software, based on the fitted models, to identify the most favorable CS:M blend by maximizing the overall desirability function.

### 2.2 Storage Stability and Antioxidant Comparison

The optimized blend was produced in three variants—negative control (100 % cassava, no antioxidant), positive control (25:75 + sodium erythorbate), and test (25:75, no erythorbate)—in triplicate. Samples were vacuum‐packed and stored at 5°C for 90 days, and analyzed at days 0, 30, 60, and 90. A 7‐day pause preceded sensory sessions to validate microbiological safety.

#### 2.2.1 pH and Color

pH: measurements were performed in triplicate using a Testo 250 pH meter (BR), previously calibrated with standard buffer solutions at pH 4.0 and 7.0. The electrode was inserted directly into the core of the mortadella samples.

Color: objective color was determined using a HunterLab MiniScan colorimeter (EZ 65/10, USA), calibrated with a white standard tile (L* = 92.8, a* = 0.9, b* = 1.1). The total color difference (Δ*E*) was calculated according to the CIE76 Equation (4):

(4) $$\Delta E=\sqrt{{\left({\Delta L}^{*}\right)}^{2}+{\left({\Delta a}^{*}\right)}^{2}+{\left({\Delta b}^{*}\right)}^{2}}\;$$

#### 2.2.2 Water‐Holding Capacity (WHC)

The WHC was determined following the method adapted from Silva Sobrinho (1999). For this analysis, 5 g cubes of the sample were placed between filter papers and subjected to a 10 kg load for 5 min. After compression, the water loss was calculated by measuring the weight difference between the initial and final sample, reflecting the amount of water retained by the sample.

#### 2.2.3 Texture Profile Analysis

The evaluation of the textural properties of the mortadellas was performed using the Texturometer XTPlus (Stable Micro Systems, UK), equipped with a 35 mm diameter shear probe. The procedure followed standard methodologies adapted for this type of product. The samples were collected during the sausage manufacturing process at the time of packaging (0 days) and stored under refrigeration at 4°C until the analyses were performed, which occurred after 30, 60, and 90 days of storage. Before being analyzed, the samples were thawed for 24 h at room temperature.

For the texture evaluation, three independent measurements were taken for each sample, with three repetitions for each formulation at each analysis point, totaling 27 samples per point. The compression test was conducted using a shear probe, obtaining the parameters of hardness, adhesiveness, elasticity, cohesiveness, and chewiness. Hardness was measured as the maximum force recorded during compression, expressed in Newtons (N), while adhesiveness indicated the force required to separate the sample from a contact surface after compression, expressed in Newton‐seconds (N s). Elasticity was determined by the sample's ability to return to its original shape after compression, and cohesiveness by the sample's integrity during the process. Chewiness was calculated as a combination of hardness and cohesiveness, reflecting the energy required to chew the product.

#### 2.2.4 Lipid Oxidation

Lipid oxidation was evaluated using the thiobarbituric acid reactive substances (TBARS) assay, which estimates the formation of secondary oxidation products, particularly malondialdehyde (MDA). The methodology was adapted from Lemon (2001). Briefly, 5.0 g of the mortadella sample was homogenized with 25 mL of 7.5% trichloroacetic acid (TCA) containing 0.01% butylated hydroxytoluene (BHT) and 0.1% EDTA to inhibit further oxidation during processing. The homogenate was centrifuged at 4000 × *g* for 10 min at 4°C, and the clear supernatant was collected.

An aliquot of 2 mL of the filtrate was mixed with 2 mL of 0.02 mol L^−1^ thiobarbituric acid (TBA). The reaction mixture was incubated in a water bath at 95°C for 40 min, then cooled in an ice bath to room temperature. The absorbance of the pink chromogen (MDA‐TBA complex) was measured at 532 nm using a UV‐Vis spectrophotometer.

Quantification was performed using a standard curve of 1,1,3,3‐tetraethoxypropane (TEP), prepared in the range of 0–10 µmol MDA kg^−1^, with a determination coefficient (*R* ^2^) greater than 0.99. The method detection limit (MDL) was 0.02 mg MDA kg^−1^, ensuring high sensitivity. All analyses were conducted in triplicate, and results were expressed as mg MDA kg^−1^ of the sample.

To ensure data reliability, all glassware was pre‐rinsed with acetone and dried at 105°C to avoid contamination. Internal quality controls, including blanks and known MDA standards, were run with each batch of samples.

#### 2.2.5 Flash Profile Sensory Analysis

Before the sensory analysis, all optimized mortadella samples (with and without antioxidants) were subjected to microbiological screening to ensure safety, following the methodology described in item 2.1.1. The sensory analysis was conducted in the Sensory Analysis Laboratory at the Federal Technological University of Paraná, Campus Campo Mourão, with 16 untrained assessors with a mean age of approximately 20 years.

The flash profile method was chosen for this sensory analysis due to its efficiency in quickly identifying key sensory attributes with a panel of untrained assessors. This technique is particularly suitable when the objective is to assess distinct sensory characteristics without requiring intensive training, as is often the case with methods like quantitative descriptive analysis (QDA). The flash profile allows for rapid identification of sensory differences between the mortadella samples, making it ideal for this exploratory study. Unlike QDA, which requires extensive training to develop a precise and consistent sensory vocabulary, flash profile enables an efficient and effective evaluation with minimal training, making it well‐suited to the panel of untrained assessors.

Assessors participated in a one‐hour orientation session focused on attribute generation and the use of unstructured line scales. Initially, two commercial mortadella samples with distinct sensory characteristics were provided, cut into cubes of approximately 1.5 cm per side. Assessors, aided by water as a neutralizing sample, were instructed to identify sensory attributes that distinguished the two products. These attributes were then discussed with the analyst to consolidate the evaluation vocabulary.

Following this phase, each judge received four randomized samples of the optimized mortadella with antioxidants and four without, across two evaluation sessions. All samples were cut into uniform cubes (∼1.5 cm^3^), coded with three‐digit random numbers, and presented monadically in randomized order, accompanied by water for palate cleansing. Assessors ranked the samples based on the intensity of the previously identified sensory attributes.

After the evaluations, data were processed using Generalized Procrustes Analysis (GPA) with the aid of Statistica 13.0, Origin 2023b, Matlab R2023b, and the Gamma application (Galvan and Bona 2024), ensuring robust multivariate analysis of the sensory profiles.

### 2.3 Statistical Analysis

Statistical analysis was conducted to assess the impact of variables on the physicochemical characteristics of the samples, including pH, WHC, lipid oxidation, texture, and objective color (L*, a*, b*, and Δ*E*), as well as the results of microbiological and sensory analyses. The analysis was performed using the Statistica 13.0 software (StatSoft Inc., USA, 2011), fitting the mean response values to a second‐order polynomial model for more precise data interpretation.

To ensure statistical robustness, all fixed and random effects were considered. Fixed effects included variables related to treatment and formulation, while random effects accounted for intrinsic variations among samples. Relevant interactions between fixed effects were explored to identify combinations of factors significantly influencing the results.

Model evaluation was performed through analysis of variance (ANOVA) with a design appropriate to the sample type, establishing statistical significance at *p* < 0.05.

### 2.4 Study Limitations and Methodological Considerations

Although the present study provided a comprehensive evaluation of the physicochemical, sensory, and antioxidant stability of mortadella formulations containing CS and Peruvian maca flour, certain analyses suggested by the literature were not included due to practical constraints.

Microstructural analysis (e.g., light microscopy or scanning electron microscopy) to locate starch granules or fat globules was not performed. This limitation is acknowledged, and future studies are encouraged to include such techniques to better understand the structural role of maca flour in meat matrices.

Only secondary lipid oxidation (TBARS) was measured during storage. Although PV is an important marker of primary oxidation, resource limitations precluded its inclusion. Nevertheless, TBARS results provided sufficient comparative trends to assess oxidative stability among treatments.

The fatty acid composition was not determined over the storage period, as the formulations were primarily designed to assess functional and sensory characteristics rather than nutritional shifts. Investigating lipid profile changes could be an interesting direction for future research, especially if the antioxidant mechanisms of maca flour are to be fully elucidated.

While all products were microbiologically screened before sensory testing to ensure safety, full shelf‐life microbiological tracking (e.g., total viable count—TVC) was not carried out during the 90‐day storage period. This is acknowledged as a limitation, and future studies should incorporate microbial dynamics to fully validate storage stability.

## 3 Results and Discussion

### 3.1 Optimization of Mortadella Formulations Incorporating CS and Peruvian Maca Flour

To support the optimization of mortadella formulations containing CS and Peruvian maca flour (M), physicochemical, microbiological, and sensory analyses were conducted. Below is an in‐depth, comprehensive discussion integrating these results, contextualized with a broad review of recent literature and considering industrial applications.

Table 2 presents the physicochemical characteristics of the emulsified meat formulations (F1–F7), which varied in the proportion of Peruvian maca flour and CS. Significant differences (*p* < 0.05) were observed in pH, WHC, and color parameters (L*, a*, b*, and Δ*E*), influenced by the type and proportion of added starches.

**TABLE 2:** Physicochemical properties of emulsified meat formulations (F1–F7).

			Color parameters			
Experiments	pH	WHC	L*	a*	b*	Δ*E*
**F1**	6.08^b^ ± 0.00	92.75^c^ ± 0.17	63.67^b^ ± 0.16	11.31^bc^ ± 0.08	11.94^a^ ± 0.24	0.00
**F2**	6.14^ab^ ± 0.00	95.87^a^ ± 0.51	67.13^a^ ± 0.30	12.66^a^ ± 0.08	10.02^b^ ± 0.21	0.96
**F3**	5.99^c^ ± 0.03	93.72^bc^ ± 0.31	66.79^ac^ ± 0.37	10.24^c^ ± 0.49	10.07^b^ ± 0.44	1.36
**F4**	6.17^a^ ± 0.00	95.27^ab^ ± 0.22	66.24^ac^ ± 0.19	11.90^ab^ ± 0.13	10.17^b^ ± 0.14	2.82
**F5**	6.08^b^ ± 0.03	94.15^bc^ ± 0.22	65.00^bc^ ± 0.69	11.30^bc^ ± 0.22	11.09^ab^ ± 0.32	4.65
**F6**	6.10^ab^ ± 0.00	94.86^ab^ ± 0.16	65.36^abc^ ± 0.37	11.90^ab^ ± 0.21	11.40^a^ ± 0.02	6.25
**F7**	6.09^b^ ± 0.01	94.74^ab^ ± 0.59	65.08^bc^ ± 0.27	11.63^ab^ ± 0.05	11.15^bc^ ± 0.15	7.79

*Note*: Values within the same column followed by different superscript letters differ significantly, as indicated by Tukey's test (*p* < 0.05); superscripts denote Tukey's grouping. Formulations F1 (0:100), F2 (100:0), F3 (25:75), and F4 (75:25) represent varying proportions of cassava starch to Peruvian maca flour, while F5–F7 (50:50) correspond to central point replicates. Color difference values (Δ*E*) were calculated using the CIELAB formula.

The pH values ranged from 5.99 (F3) to 6.17 (F4), aligning with the desirable range for emulsified meat products, as values around 6.0 are associated with improved water‐holding capacity and emulsion stability (Puolanne et al. 2001; Jang & Chin, 2011). These values were also similar to those observed in traditional meat formulations using whey protein or ovine meat (Terra et al. 2009; Guerra et al. 2012). The relatively stable pH may be attributed to the buffering effect of maca flour, which contains amino acids and various bioactive compounds (Gonzales 2012), contributing to chemical equilibrium during processing.

Regarding WHC, all formulations presented high WHC values, ranging from 92.75% (F1) to 95.87% (F2). F2, composed entirely of CS, showed the highest WHC, highlighting the hydrophilic potential of starches. However, formulations with higher proportions of maca flour, such as F4 (75:25) and F5 (50:50), also exhibited elevated WHC. This finding corroborates Zhang et al. (2020), who observed that plant‐based flours with high dietary fiber content tend to retain more water due to their structural and chemical properties. The concurrent presence of proteins and carbohydrates (from maca and cassava) may synergistically enhance WHC, supporting the mechanisms proposed by Lucarini et al. (2020) and Hughes et al. (2014). In addition, the use of a thermoplastic casing likely contributed to the retention of WHC during thermal processing, a factor not considered in studies such as Nascimento et al. (2008), where WHC ranged from 39.91 to 50.37%.

In terms of color, the lightness (L*) of the mortadella ranged from 63.67 (F1) to 67.13 (F2), higher than the L* values commonly reported in the literature (Barbosa et al. 2006; Matos et al. 2007). This may result from the cooking method used, reaching 68°C in a water bath within 45 min, which tends to yield lighter‐colored products. The redness index (a*) ranged from 10.24 to 12.66, generally lower than values reported by Barbosa et al. (22.00) and Matos et al. (12.5), potentially due to the reduced time for pink color development. The yellowness index (b*) varied from 10.02 to 11.94, with the highest value observed in F1, composed entirely of maca flour. This result supports the hypothesis that the natural yellow pigments in maca (such as carotenoids) influenced color expression (Da Silva Leitão Peres et al. 2020).

Color difference (Δ*E*) values ranged from 0.00 (F1) to 7.79 (F7), indicating visually perceptible differences among formulations, especially in the central point replicates (F5–F7), which suggest greater color heterogeneity. Although these color variations were measurable and perceptible, sensory evaluation in subsequent stages indicated that such differences did not negatively impact consumer acceptance. These findings demonstrate that incorporating maca flour into emulsified meat products improves WHC and modifies color characteristics while maintaining ideal pH levels. Such technological advantages, combined with potential nutritional benefits, support the feasibility of using maca as a functional ingredient in meat product reformulation.

Similar reformulations using plant‐based ingredients in emulsified meat products have shown varying effects depending on the botanical source. For instance, the incorporation of chia flour has been reported to improve water‐holding capacity and enhance nutritional value due to its high fiber and omega‐3 content (Pintado et al. 2016; Serdaroğlu et al. 2024). Likewise, ingredients such as beetroot powder, spinach extract, and grape seed flour have been used to enrich products with natural antioxidants, improving oxidative stability and shelf life (Domínguez et al. 2019). Compared to these ingredients, maca flour demonstrated similar or superior performance in maintaining high WHC and contributing to distinct color characteristics due to its pigment profile. These findings highlight maca's potential as a multifunctional additive, capable of promoting both technological and functional improvements in emulsified meat matrices.

Table 3 shows the microbiological results of the emulsified meat formulations. All samples presented microbial counts within the acceptable limits established by current legislation. However, the detection of *Staphylococcus* spp. and *Clostridium* spp. in some formulations, particularly F7, which showed 4.00 log CFU g^−1^, warrants critical attention. Although these values comply with microbiological standards, they may reflect deficiencies in hygienic handling, thermal processing, or equipment sanitation during production. Even within permissible limits, the presence of these microorganisms could compromise product shelf life and raise concerns regarding potential risks if good manufacturing practices are not rigorously enforced.

**TABLE 3:** Microbiological counts of the samples and presence of *Salmonella* spp.

Experiments	Total aerobic mesophilic bacteria	Thermotolerant coliforms (45°C)	*Escherichia coli*	Coag.+ *Staphylococcus*	Sulfite‐reducing *Clostridium* spp.	*Salmonella* spp. (25 g)
F1	2.15 ± 0.10	<1.0	Not detected	<1.0	<1.0	Absent
F2	2.30 ± 0.12	<1.0	Not detected	<1.0	<1.0	Absent
F3	2.20 ± 0.09	<1.0	Not detected	<1.0	3.03 ± 0.18	Absent
F4	2.05 ± 0.11	<1.0	Not detected	<1.0	<1.0	Absent
F5	2.18 ± 0.13	3.33 ± 0.58	Not detected	<1.0	<1.0	Absent
F6	2.25 ± 0.10	<1.0	Not detected	2.61 ± 0.03	<1.0	Absent
F7	2.12 ± 0.08	<1.0	Not detected	4.00 ± 0.00	<1.0	Absent
**Regulatory limit**	–	3.00	Absence	3.48 (*≈*3 × 10^3^)	2.70 (*≈*5 × 10^2^)	Absence

*Note*: Formulations F1 (0:100), F2 (100:0), F3 (25:75), and F4 (75:25) represent varying proportions of cassava starch to Peruvian maca flour, while F5–F7 (50:50) correspond to central point replicates.

Values are expressed as mean ± standard deviation (*n* = 3).

<1.0 indicates microbial counts below the detection limit of the method (1.0 log CFU g^−1^).

Not detected refers to the absence of *E. coli* in 1 g of sample.

Absent refers to the absence of *Salmonella* spp. in 25 g of sample.

Regulatory limits are based on Brasil (2003) standards for ready‐to‐eat meat products.

*Salmonella* spp. was absent in 25 g of all samples, and *Escherichia coli* was not detected. Most microbial counts, including thermotolerant coliforms, *Staphylococcus aureus*, and sulfite‐reducing *Clostridium* spp., remained below the detection limit (<1.0 log CFU g^−1^) in the majority of samples. However, measurable levels were observed in a few cases: F3 showed 3.33 ± 0.58 log CFU g^−1^ of *Clostridium* spp., F5 had 3.33 ± 0.58 log CFU g^−1^ of thermotolerant coliforms, F6 showed 2.61 ± 0.03 log CFU g^−1^ of coagulase‐positive *Staphylococcus*, and F7 had 4.00 ± 0.00 log CFU g^−1^ of coagulase‐positive *Staphylococcus*. Nevertheless, all values remained within the acceptable regulatory limits.

These results reflect the adoption of effective hygienic practices during preparation and processing, as well as the efficacy of thermal treatment in microbial control. Maintaining low microbial loads is essential not only for food safety but also for shelf‐life extension. According to Huang et al. (2020), such microbiological quality is fundamental in ready‐to‐eat meat products, as microbial spoilage and contamination are among the primary causes of product rejection and economic loss. In this context, the use of thermoplastic casings and the controlled heating protocol likely contributed to minimizing microbial survival, ensuring a microbiologically stable product throughout its storage.

The results of the acceptance test are presented in Table 4. Tukey's test was applied at a 5% significance level to evaluate whether there were statistical differences among the means of the sensory attributes across the seven formulations. The analysis of variance (ANOVA) indicated that no significant differences (*p* > 0.05) were found among the samples for aroma, color, flavor, texture, or overall impression.

**TABLE 4:** Average acceptance ratings and variability of sensory attributes for F1–F7.

Experiments	Aroma	Color	Flavor	Texture	Overall impression
**F1**	6.92^a^ ± 0.17	6.00^a^ ± 0.18	6.94^a^ ± 0.18	6.86^a^ ± 0.20	6.79^a^ ± 0.17
**F2**	7.04^a^ ± 0.16	6.51^a^ ± 0.17	7.14^a^ ± 0.15	6.69^a^ ± 0.17	7.07^a^ ± 0.13
**F3**	7.20^a^ ± 0.14	6.31^a^ ± 0.18	7.31^a^ ± 0.14	6.91^a^ ± 0.19	7.16^a^ ± 0.14
**F4**	7.15^a^ ± 0.15	6.65^a^ ± 0.16	7.22^a^ ± 0.16	6.89^a^ ± 0.17	7.14^a^ ± 0.14
**F5**	6.98^a^ ± 0.15	6.15^a^ ± 0.17	7.18^a^ ± 0.16	6.53^a^ ± 0.20	7.19^a^ ± 0.15
**F6**	7.18^a^ ± 0.14	6.49^a^ ± 0.15	6.91^a^ ± 0.18	6.99^a^ ± 0.16	7.09^a^ ± 0.15
**F7**	7.02^a^ ± 0.16	6.54^a^ ± 0.16	7.14^a^ ± 0.15	6.71^a^ ± 0.17	7.12^a^ ± 0.16

*Note*: Values within the same column followed by different superscript letters differ significantly, as indicated by Tukey's test (*p* < 0.05); superscripts denote Tukey's grouping. Formulations F1 (0:100), F2 (100:0), F3 (25:75), and F4 (75:25) represent varying proportions of cassava starch to Peruvian maca flour, while F5–F7 (50:50) correspond to central point replicates.

The results suggest that the partial or total substitution of CS with Peruvian maca flour, in the tested proportions, did not negatively affect the sensory acceptability of the emulsified meat products. All formulations received scores above 6.0 on the 9‐point hedonic scale, indicating general consumer satisfaction across attributes.

Although no significant differences were found, formulations F4 and F5 exhibited slightly higher mean scores for flavor and texture. This trend may be attributed to the enhanced WHC observed in these samples, likely improving juiciness and mouthfeel, key factors influencing consumer preference. In addition, maca flour is known to contain amino acids and secondary metabolites associated with umami flavor perception (Wang et al. 2023), which may have subtly enhanced the palatability of these formulations.

Regarding color acceptance, which is often influenced by visual expectations, all formulations remained consistent despite the inclusion of maca flour, which imparts a slightly darker, yellowish hue. This consistency may reflect an increasing consumer acceptance, or even preference, for products containing visible cues of natural and functional ingredients, as highlighted by Reis et al. (2023).

The mean scores for aroma ranged from 6.92 to 7.20, for color from 6.00 to 6.54, for flavor from 6.94 to 7.31, for texture from 6.69 to 6.98, and for overall impression from 6.79 to 7.17. Most of these values were close to 7, which corresponds to “moderately liked” on the hedonic scale.

These results compare favorably with those reported by Tavares et al. (2007), who obtained an average of 5.85 in the acceptance test for rabbit meat burgers. Similarly, Nassu, Silva, et al. (2002) found no significant differences (*p* > 0.05) across treatments for fermented goat meat sausages, with overall acceptance and other attributes such as appearance, aroma, flavor, and texture scoring between 5 and 6. Pedroso and Demiate (2008) also observed no significant differences in acceptability for cooked turkey ham, where scores ranged from 6 to 7. These comparisons further underscore the favorable sensory performance of formulations containing maca flour in the present study.

In terms of sensory attributes, maca flour demonstrated favorable performance, especially in formulations with balanced ratios (e.g., F4 and F5), which maintained desirable texture, color, and flavor profiles. Similar sensory acceptance has been reported in emulsified products reformulated with quinoa, lentil, or rice flour (Choi et al. 2016; Ismail et al. 2020), although these often require masking agents to address off‐flavors. In contrast, maca's naturally sweet and nutty profile may enhance palatability without compromising sensory quality, positioning it as a promising clean‐label ingredient for functional reformulations.

Regarding sensory evaluation, formulations with maca flour, especially those containing 50% or more maca flour, struck a favorable balance between texture, flavor, and color. The sensory results aligned with previous studies involving other plant‐based ingredients, such as quinoa and rice flour, which have also been accepted in emulsified meat products (Jiménez‐Colmenero et al. 2010; Alvarado et al. 2017). The inclusion of maca flour in formulations like F4 (75:25) and F5 (50:50) resulted in products that were notably more accepted than those with alternative starches, suggesting that maca flour not only enhances the nutritional value but also positively contributes to the overall sensory profile, thereby improving consumer appeal. In contrast, other plant‐based flours such as lentil and chickpea flour have been reported to sometimes lead to undesirable textures and off‐flavors, a challenge that maca flour effectively mitigates (Moreno et al. 2021).

Table 5 shows the regression coefficients obtained from the mathematical modeling of the dependent variables as a function of the proportions of CS and maca flour (M), including the interaction term (CS×M) when significant. The models revealed acceptable coefficients of determination (*R* ^2^), indicating a good fit to the experimental data, especially for the sensory variables, where the global impression reached *R* ^2^ = 0.84.

**TABLE 5:** Regression coefficients for the dependent variables analyzed in the experimental design.

Variable	Intercept	β_1_ (cassava starch)	β_1_ (maca flour)	β_12_ (Interaction CS×M)	*R* ^2^
pH	–	6.11	6.08	–	0.45
WHC (g 100 g^−1^)	–	93.54	95.42	–	0.55
L* (lightness)	–	64.11	67.11	–	0.65
a* (redness)	–	11.36	11.77	–	0.57
b* (yellowness)	–	11.62	10.05	–	0.61
Overall impression	–	6.96	7.20	+0.86	0.84

These models served as the basis for generating the desirability function, which aims to maximize product quality by simultaneously optimizing multiple responses. The variables selected for this optimization included pH, WHC, color parameters (L*, a*, b*), and overall impression. Each of these was individually modeled, and their combination through the desirability function allowed the identification of the formulation that best met all quality criteria.

It is worth noting that the inclusion of color parameters in the desirability optimization is justified by the direct impact of CS and maca flour on the color characteristics of the final product. In addition, pH and WHC are crucial physicochemical markers of protein functionality and product stability, while the overall impression reflects the sensory acceptability of the final formulation.

**FIGURE 1:**
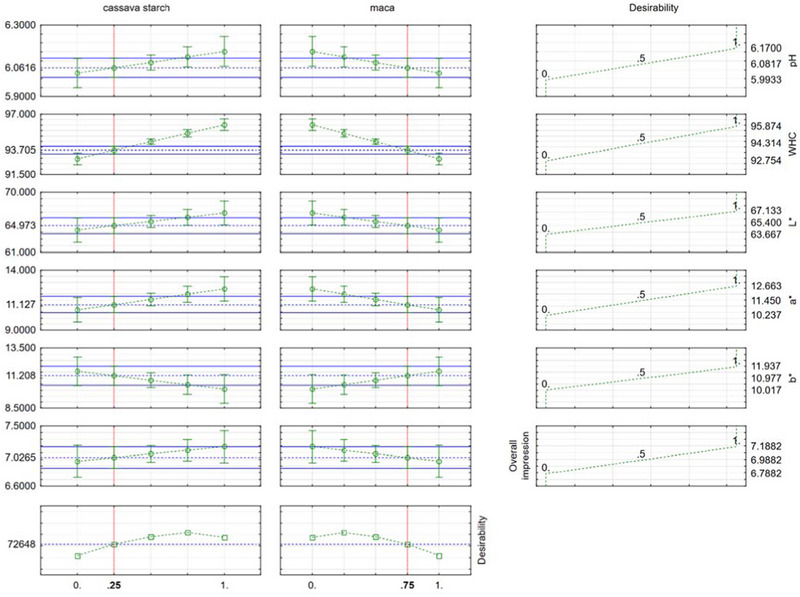
Optimized mixture of cassava starch and maca flour according to the desirability function.

Based on the desirability function (Figure 1), the optimization process suggested a formulation of 25% CS and 75% maca flour, yielding a global desirability index of 0.73. This formulation represents the best balance between the technological and sensory parameters analyzed. It maximized the desirability function, selecting a balanced blend that ensured desirable characteristics in all evaluated aspects. This optimized blend is consistent with previous studies, which suggest that higher starch concentrations improve water retention and color lightness, while a moderate addition of maca contributes bioactive compounds and sensory appeal without compromising technological functionality.

Furthermore, incorporating overall sensory impression as a variable in the optimization process ensured that the selected formulation was not only technologically effective but also organoleptically preferred. This integrative approach aligns with recent product development frameworks (Costa et al. 2020; Savadkoohi et al. 2014), emphasizing the importance of multi‐criteria optimization in the development of clean‐label meat analogs.

### 3.2 Evaluation of the Optimized Mortadella Formulation

This stage aimed to evaluate the storage stability and antioxidant efficacy of the optimized formulation over a 90‐day period. Two trials were developed: one with the optimized formulation, including the synthetic antioxidant sodium erythorbate, and another with the optimized formulation, without adding the synthetic antioxidant. Table 6 presents the results of the physicochemical analyses conducted throughout this period. These analyses include average pH values, WHC, objective color measurements, and lipid oxidation levels of the optimized mortadella formulation, which consisted of 25% CS and 75% Peruvian maca flour.

**TABLE 6:** Averages of the physicochemical analysis values for the optimized formulations with and without the addition of the synthetic antioxidant (sodium erythorbate).

Experiments	pH	WHC (g 100 g^−1^)	Color parameters			Lipid oxidation (mg MDA kg^−1^)
			L*	a*	b*	
**With antioxidant (Day 0)**	5.99^c^ ± 0.01	95.24^a^ ± 0.30	64.35^f^ ± 0.06	13.35^a^ ± 0.10	11.96^ac^ ± 0.18	1.75^c^ ± 0.00
**With antioxidant (Day 30)**	6.01^b^ ± 0.00	94.25^a^ ± 0.48	64.42^ef^ ± 0.06	12.51^ab^ ± 0.03	11.26^be^ ± 0.04	3.91^a^ ± 0.01
**With antioxidant (Day 60)**	6.04^b^ ± 0.01	94.34^a^ ± 0.76	65.44^ab^ ± 0.13	11.57^b^ ± 0.01	11.85^cd^ ± 0.04	1.87^c^ ± 0.10
**With antioxidant (Day 90)**	6.02^bc^ ± 0.02	94.05^a^ ± 0.54	64.90^cd^ ± 0.04	11.48^b^ ± 0.04	11.43^de^ ± 0.03	2.10^bc^ ± 0.23
**Without antioxidant (Day 0)**	6.05^ab^ ± 0.01	93.54^a^ ± 0.31	64.52^cde^ ± 0.04	12.04^b^ ± 0.02	10.78^cd^ ± 0.01	2.68^c^ ± 0.02
**Without antioxidant (Day 30)**	6.01^bc^ ± 0.01	95.45^a^ ± 0.35	64.82^def^ ± 0.20	11.78^ab^ ± 0.77	11.87^b^ ± 0.21	1.48^b^ ± 0.01
**Without antioxidant (Day 60)**	6.08^a^ ± 0.00	94.11^a^ ± 0.93	65.76^a^ ± 0.02	12.11^ab^ ± 0.04	12.44^a^ ± 0.01	3.51^a^ ± 0.31
**Without antioxidant (Day 90)**	6.07^a^ ± 0.01	93.25^a^ ± 0.94	65.26^bc^ ± 0.07	11.92^b^ ± 0.03	11.31^e^ ± 0.03	2.59^b^ ± 0.12

*Note*: Identical superscripts in the same column for the same parameter indicate means with no statistically significant difference (*p* > 0.05) in the Tukey test.

For the data presented in Table 6, the pH analyses were within the average range for this type of meat product. They showed values close to each other, and almost all showed no significant differences. The water retention capacity was similar among the eight samples. This water retention capacity in the products is primarily due to the properties of the myofibrillar proteins, the type of thermoplastic casing used, and other discussions previously presented that were repeated in the optimized formulation tests. The addition of other plant ingredients, such as starch, soy protein, and, in the case of this study, maca flour, helps the emulsion retain water and remain stable during cooking, as well as improving texture, tenderness, slicing ability, yield, among other important properties for meat products (Honikel 1998).

Regarding objective color, the highest luminosity was found in the sample without an antioxidant after 60 days of shelf life. The a* color parameter showed higher values with no significant differences for samples with antioxidants at 0 and 30 days and for samples without antioxidants at 30 and 60 days. The other samples did not show differences between the means. The samples with higher values of the b* color parameter were those with antioxidants at 0 days and without antioxidants at 60 days. For the other samples, there were significant differences among all means for this objective color parameter.

The variation in color during storage was assessed by calculating the total color difference (Δ*E*) between sampling days for formulations with and without antioxidants. In samples containing antioxidants, Δ*E* values ranged from 1.10 between Day 0 and Day 30 to a peak of 1.51 between Days 30 and 60, before decreasing to 0.69 from Day 60 to Day 90. This pattern indicates minimal and likely imperceptible visual changes overall, with improved color stability particularly evident after the first month of storage.

Samples without antioxidant addition exhibited slightly higher and more consistent color variability, with Δ*E* values of 1.16, 1.15, and 1.25 across the same intervals. Although these values remained below the commonly accepted perceptibility threshold of 2.3, the gradual upward trend suggests a continuous, albeit subtle, color shift throughout storage in the absence of antioxidants.

Comparing the antioxidant‐treated samples at Day 90 to non‐antioxidant samples at day 0, an Δ*E* of 0.94 was observed, reinforcing the role of antioxidants in preserving initial color characteristics over time. Importantly, the unique hue contributed by maca flour did not negatively impact consumer expectations or acceptance, as the color variations remained within acceptable visual limits.

Overall, while the color differences were analytically measurable, they were unlikely to be perceptible to consumers and did not compromise the product's marketability. These results highlight the functional benefit of antioxidant addition in enhancing color consistency and shelf life in emulsified meat products like mortadella.

**FIGURE 2:**
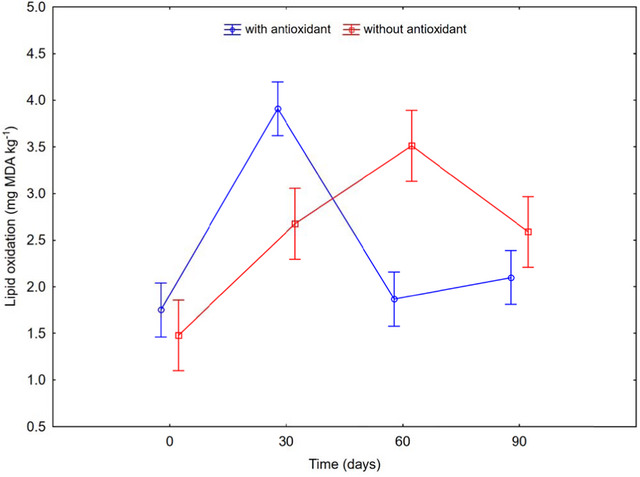
Relationship between lipid oxidation and shelf life.

Lipid oxidation in meat products can exhibit fluctuations due to several factors, including the presence of antioxidants, storage conditions, and the intrinsic properties of the formulation. According to Bogolyubova (2022), lipid oxidation is a major cause of deterioration in meat products, affecting sensory quality and nutritional value, and negatively impacting consumer acceptability. Figure 2 shows that lipid oxidation values increased for samples with antioxidants between 0 and 30 days and again from 60 to 90 days, with the highest peak at 30 days. This pattern may be explained by the initial effectiveness of antioxidants, which could diminish over time, leading to temporary stabilization of lipid oxidation between 30 and 60 days.

Conversely, samples without antioxidants exhibited an increase in lipid oxidation from 0 to 60 days, peaking at 60 days, followed by a decrease at 90 days. The peak at 60 days can be attributed to the lack of antioxidants, which allows for greater lipid degradation. The subsequent decrease might result from the depletion of oxidizable lipids or the formation of secondary oxidation products that are less reactive. According to Al‐Kahtani et al. (1996), lipid oxidation values should be below 3.0 mg MDA kg^−1^ for proper preservation. In this study, the optimized formulation (25% CS and 75% maca flour) maintained lipid oxidation levels below this threshold, indicating adequate preservation and stability.

These results demonstrate that while antioxidant presence and storage conditions significantly influence lipid oxidation, leading to observed fluctuations, the optimized formulation remains effective in preserving the product with satisfactory quality. Furthermore, the color stability observed through Δ*E* values supports these findings: samples with antioxidants showed relatively low Δ*E* values throughout storage, especially between days 60 and 90 (Δ*E* = 0.69), indicating minimal visual degradation. In contrast, the samples without antioxidants showed more variable and slightly higher Δ*E* values across the same period, suggesting that oxidative processes, including lipid oxidation, contributed to perceptible color changes. This correlation highlights the role of antioxidants not only in lipid preservation but also in maintaining the visual quality of the product, which is essential for consumer acceptance.

Observing the results presented in Table 7 of the second stage of this work, for texture parameters, it is possible to see significant differences between the samples in terms of hardness, chewiness, and cohesiveness. For hardness, the samples with antioxidants at 30 and 60 days of shelf life did not show significant differences, just as the samples with 90 days did, regardless of whether the antioxidant was added or not. These values are also corroborated by chewiness, which is directly related to hardness—i.e., the greater the hardness, the greater the chewiness, and vice versa. The samples with the highest values for these two parameters were those with the antioxidant at time 0, while the lowest values were found in samples without the antioxidant at 90 days of shelf life. Maqsood et al. (2012) evaluated the addition of tannic acid as an antioxidant agent in meat product formulations. They observed that the control (without tannic acid) showed a texture softening after 20 days of storage at 4°C. It was concluded that this behavior was likely due to the proteolytic action promoted by bacterial protease enzymes due to product aging, and this behavior, particularly in hardness (N), was observed over time and aging in both optimized formulations (Table 7). For adhesiveness and elasticity parameters, there were no differences between the means. For cohesiveness, the lowest value was determined for the sample with antioxidants at time 0, and the highest value for the sample with 90 days, also with added antioxidants.

**TABLE 7:** Average texture parameters for optimized formulations with and without antioxidant.

Experiments	Hardness (N)	Adhesiveness (N s)	Elasticity (mm)	Chewiness (N mm)	Cohesiveness
**With antioxidant (Day 0)**	75.08^a^ ± 5.52	−0.17^a^ ± 0.04	0.92^a^ ± 0.01	5354.64^a^ ± 327.04	0.77^e^ ± 0.01
**With antioxidant (Day 30)**	51.34^bc^ ± 3.30	−0.23^a^ ± 0.04	0.92^a^ ± 0.02	3773.21^bc^ ± 182.12	0.79^cde^ ± 0.01
**With antioxidant (Day 60)**	52.89^bc^ ± 2.61	−0.14^a^ ± 0.04	0.94^a^ ± 0.02	4020.05^abc^ ± 186.22	0.80^cde^ ± 0.01
**With antioxidant (Day 90)**	37.17^c^ ± 2.40	−0.15^a^ ± 0.05	0.96^a^ ± 0.01	3030.85^c^ ± 182.95	0.84^a^ ± 0.01
**Without antioxidant (Day 0)**	69.27^ab^ ± 8.41	−0.15^a^ ± 0.03	0.91^a^ ± 0.02	4928.36a^b^ ± 497.49	0.78^de^ ± 0.01
**Without antioxidant (Day 30)**	44.24^c^ ± 3.84	−0.14^a^ ± 0.04	0.94^a^ ± 0.03	3386.28^c^ ± 272.21	0.80^bcd^ ± 0.01
**Without antioxidant (Day 60)**	54.17^abc^ ± 6.92	−0.12^a^ ± 0.04	0.90^a^ ± 0.02	4022.61^abc^ ± 481.69	0.82^abc^ ± 0.01
**Without antioxidant (Day 90)**	33.71^c^ ± 2.57	−0.10^a^ ± 0.03	0.95^a^ ± 0.02	2680.63^c^ ± 163.19	0.83^ab^ ± 0.01

*Note*: Identical superscripts in the same column for the same parameter indicate means with no statistically significant difference (*p* > 0.05) in the Tukey test.

The tests of the optimized formulations were subjected to sensory analysis using the flash profile method. Before the sensory analysis at each time interval, the microbiological conditions of the mortadellas were evaluated to ensure the food safety of the assessed product. Table 8 presents the results of the microbiological analyses.

**TABLE 8:** Microbiological analysis results of the optimized samples.

Experiments	Thermotolerant coliforms (45°C)	Coag.+ *Staphylococcus*	Sulfite‐reducing *Clostridium* spp.	*Salmonella* spp. (25 g)
With antioxidant (0°C)	<1.0	<1.0	<1.0	Absent
With antioxidant (30°C)	<1.0	<1.0	<1.0	Absent
With antioxidant (60°C)	<1.0	<1.0	<1.0	Absent
With antioxidant (90°C)	<1.0	<1.0	<1.0	Absent
Without antioxidant (0°C)	<1.0	<1.0	<1.0	Absent
Without antioxidant (30°C)	<1.0	<1.0	<1.0	Absent
Without antioxidant (60°C)	<1.0	4.00 ± 0.00	<1.0	Absent
Without antioxidant (90°C)	<1.0	<1.0	<1.0	Absent
**Regulatory limit**	3.00	3.48 (*≈* 3 × 10^3^)	2.70 (*≈* 5 × 10^2^)	Absence

*Note*: <1.0 indicates microbial counts below the detection limit of the method (1.0 log CFU g^−1^).

Not detected refers to the absence of *E. coli* in 1 g of sample.

Absent refers to the absence of *Salmonella* spp. in 25 g of sample.

Regulatory limits are based on Brasil (2003) standards for ready‐to‐eat meat products.

After conducting the physicochemical, microbiological, and texture analyses of the optimized mortadella formulation, with and without the addition of an antioxidant, over a storage period of 0 to 60 days, sensory evaluation was performed using the flash profile method. A panel of 16 trained assessors (J1 to J16), all over 20 years old, participated in the sensory characterization. The eight formulations analyzed were: CAOd0R1, CAOd0R2, SAOd0R1, SAOd0R2, CAOd60R1, CAOd60R2, SAOd60R1, and SAOd60R2. These were selected to capture sensory changes related to antioxidant presence and storage time.

Assessors used a range of descriptors to differentiate the samples based on texture, aroma, and visual appearance. Terms such as “fatty” were identified in some formulations and were interpreted as part of the overall sensory profile without inferring direct associations with product quality attributes.

**FIGURE 3:**
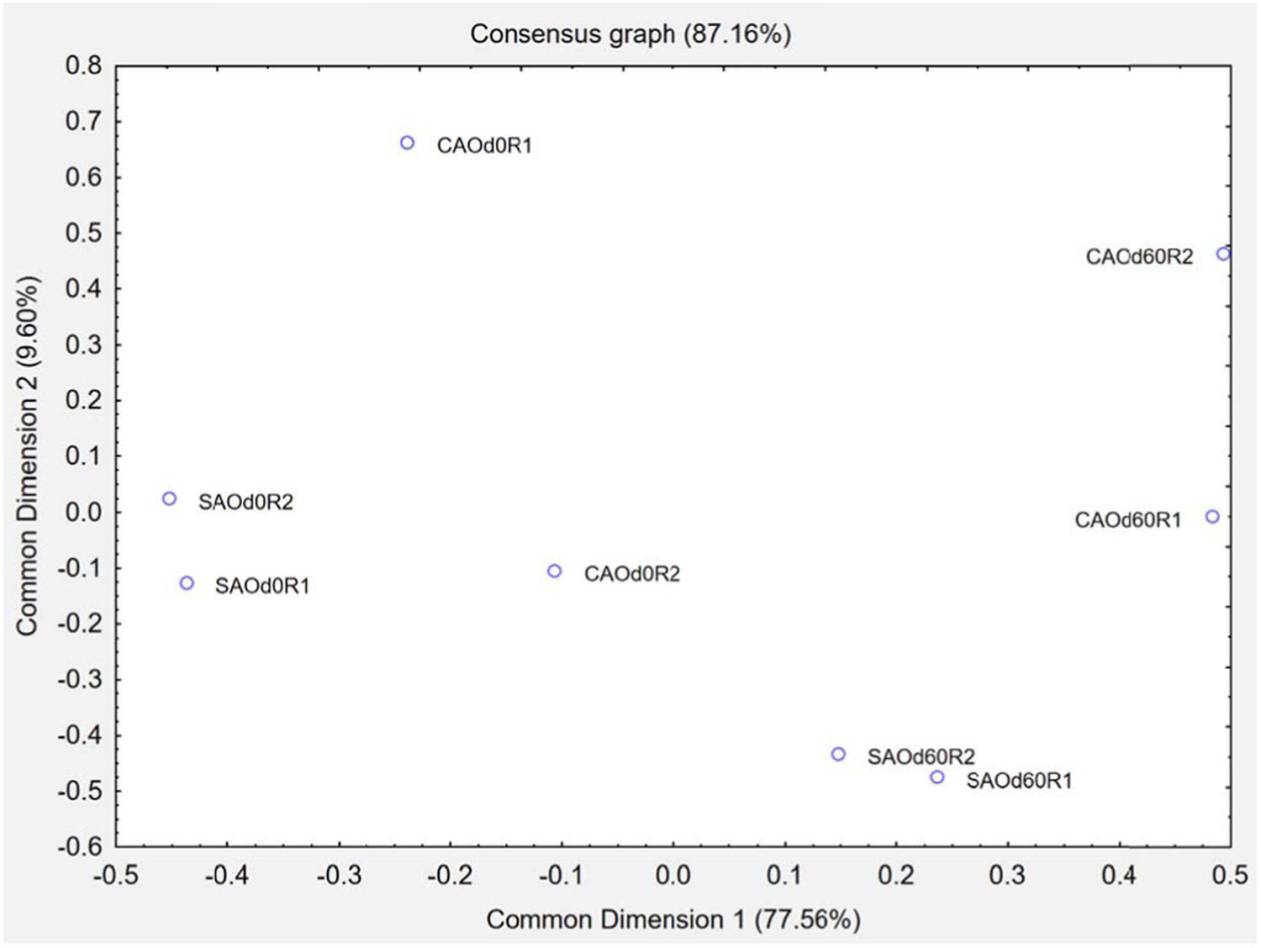
Sensory consensus of mortadella samples obtained by flash profile.

The sorting data from the flash profile were analyzed using the ComDim method, which generated a consensus configuration among assessors and identified two main sensory dimensions (CD1 and CD2), explaining 87.16% of the total variance (Figure 3).

In CD1, the most accurate segmentation was observed in the duplicate of the sample without antioxidants at Day 60, followed by without antioxidants at Day 0. This suggests that antioxidant‐free samples developed more distinguishable sensory features during storage. In contrast, samples with antioxidants (both at Day 0 and Day 60) had less agreement among assessors, indicating that these samples presented more homogeneous or less intense sensory cues.

In CD2, the highest discrimination ability was observed in the duplicate of the sample without antioxidants at Day 0, reinforcing that even short‐term storage can influence perceptibility when antioxidants are not used.

**FIGURE 4:**
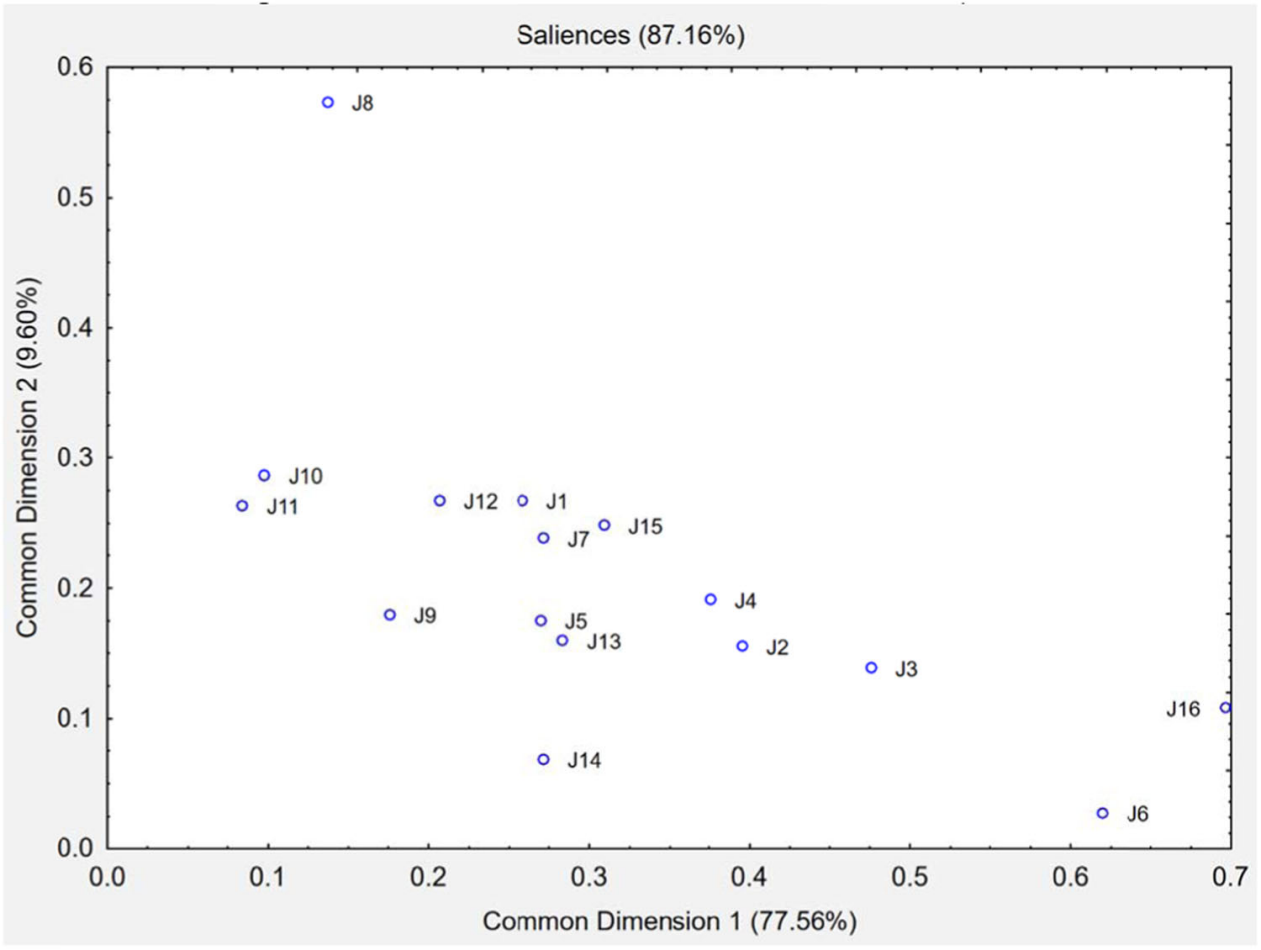
Attribute salience in flash profile of mortadella samples.

Figure 4 shows the saliences of each assessor for each common dimension, i.e., the weight associated with each assessor in the formation of each common dimension. Assessor J16 is the most important for the construction of CD1, while J8 contributes the most to the formation of CD2. The salience analysis also helps identify assessors who had difficulty discriminating the samples, i.e., those with low salience in all the most relevant common dimensions (Cariou et al. 2019). Assessors J11 and J6 had low salience values in both dimensions.

Table 9 shows the sensory attributes most strongly correlated (*p* < 0.05) with each common dimension. In CD1, negative correlations were observed with hardness, mortadella aroma, mortadella flavor, salty flavor, and uniformity, suggesting these characteristics were less intense in samples on the negative side of CD1. In contrast, positive correlations were observed with fat presence, seasoning flavor, smooth texture, shine, and pink color, suggesting a more pronounced expression of these traits in samples positioned positively along CD1.

**TABLE 9:** Significant correlations (*p* < 0.05) between common dimensions (CD) and sensory attributes identified in the Flash Profile.

Correlations CD1		Correlations CD2	
Negatives (−)	Positives (+)	Negatives (−)	Positives (+)
Hardness (1)	Opaque (1)	Fat presence (1)	Mortadella flavor (4)
Mortadella aroma (5)	Fat presence (1)	Mortadella odor (1)	Pink color (2)
Mortadella flavor (2)	Seasoning flavor (2)		Salty flavor (1)
Salty flavor (1)	Hardness (5)		Aerated texture (1)
Uniformity (1)	Smooth texture (1)		Hardness (1)
Homogeneous appearance (1)	Shine (1)		
	Pink color (5)		
	Salty flavor (1)		

In CD2, the negative quadrant was associated with fat presence, mortadella odor, and hardness, while the positive quadrant correlated with mortadella flavor, pink color, salty flavor, aerated texture, and again, hardness, reinforcing its role as a key driver of sensory perception.

Among the samples, SAOd0R1 and CAOd0R2 were located in the negative quadrants of both CD1 and CD2, indicating that these formulations were perceived as softer, less pink, and with less intense mortadella flavor, but with stronger fat presence and aroma—traits potentially linked to the absence of antioxidant and the freshness of the product.

On the other hand, samples in the positive quadrant of both dimensions, including especially CAOd60R2 and CAOd0R1, exhibited greater hardness, pink color, and a more pronounced mortadella flavor. The presence of antioxidants and storage may have played a role in preserving or enhancing these sensory attributes. Notably, CAOd60R2 had hardness as its defining attribute in CD1, with mortadella aroma being the least intense, while CAOd0R1 stood out in CD2, marked by mortadella flavor and low‐fat presence.

These findings suggest that the addition of maca flour, particularly when combined with antioxidant use and storage time, influenced key sensory dimensions such as fat presence, color, and flavor. The samples without antioxidants tended to express attributes more associated with freshness (e.g., aroma, fat perception), while those with antioxidants better retained structural and visual traits valued in mortadella, such as hardness and color intensity. This balance between sensory freshness and stability is essential when considering consumer acceptance and market positioning.

## 4 Conclusion

This study investigated the reformulation of mortadella using different proportions of Peruvian maca flour and CS, evaluating not only their effects on physicochemical, microbiological, and sensory properties but also their role in oxidative stability over a 90‐day storage period. The research contributes to the current demand for clean‐label, functional meat products and fills an important gap in the literature regarding the use of maca flour in emulsified meat systems.

As an initial step, the study conducted physicochemical analyses, which revealed that formulations containing 75% maca flour and 25% CS had consistent WHC, which is important for product texture and yield. The inclusion of maca also influenced color parameters, especially luminosity, and yellowness, reflecting the natural pigment profile of maca. Microbiological tests confirmed the safety of all formulations, and sensory acceptance remained satisfactory, even with high levels of maca flour, suggesting no negative impact on consumer perception.

In the next stage, the antioxidant effect was analyzed in formulations with and without the addition of natural antioxidants. Lipid oxidation levels, measured by the TBARS assay, remained within acceptable limits, and antioxidant‐treated samples showed greater oxidative stability over time. This was particularly evident in the optimized formulation (75% maca, 25% starch), which maintained stable color and texture characteristics throughout storage. These results demonstrate the functional contribution of maca's bioactive compounds, such as polyphenols and glucosinolates, to shelf‐life extension.

The flash profile analysis provided further insights into sensory performance. While consumer panels perceived subtle differences in attributes such as “fat presence,” “pink color,” and “mortadella flavor,” these did not compromise overall acceptance. The optimized formulation was identified as the most promising, achieving balanced performance across sensory and instrumental texture parameters. Importantly, the sensory discussion was deepened to connect these observations with the functional role of maca, enhancing the interpretation of consumer‐relevant traits.

When contextualized with existing literature, maca flour shows comparable or superior functional properties to other plant‐based flours like chickpea, quinoa, or amaranth. In addition to improving WHC and oxidative stability, maca aligns with market trends focused on natural, additive‐free ingredients and nutritional enhancement. Its use represents a viable innovation strategy for the processed meat industry.

Substituting CS with maca flour in mortadella formulations proved to be both functionally and sensorially advantageous. The antioxidant effects observed, along with the stable physicochemical and microbiological profiles, suggest that maca flour contributes to the development of cleaner‐label, high‐quality meat products with extended shelf life. These findings support the industrial application of maca flour and open new pathways for the incorporation of underutilized Andean ingredients into meat systems, with potential health, sustainability, and market benefits.

## Author Contributions

**Natália da Silva Leitão Peres**: conceptualization, investigation, methodology, project administration. **Leticia Cabrera Parra Bortoluzzi**: methodology, validation. **Flávia Aparecida Reitz Cardoso**: writing – original draft, writing – review and editing, validation, methodology. **Renata Hernandez Barros Fuchs**: methodology, validation. **Nathália Letícia Hernandez Brito**: methodology, validation. **Sahra Gadia Trelha**: methodology, validation. **Leila Larisa Medeiros Marques**: methodology, validation. **Anielle de Oliveira**: methodology, validation. **Evandro Bona**: methodology, validation. **Adriana Aparecida Droval**: conceptualization, investigation, methodology, validation.

## Conflicts of Interest

The authors declare no conflicts of interest.
